# Deep Reinforcement Learning Based Resource Allocation Strategy in Cloud-Edge Computing System

**DOI:** 10.3389/fbioe.2022.908056

**Published:** 2022-08-04

**Authors:** Jianqiao Xu, Zhuohan Xu, Bing Shi

**Affiliations:** ^1^ Department of Information Security, Naval University of Engineering, Wuhan, China; ^2^ School of Computer Science and Artificial Intelligence, Wuhan University of Technology, Wuhan, China; ^3^ Shenzhen Research Institute of Wuhan University of Technology, Shenzhen, China

**Keywords:** collaborative cloud-edge computing, resource allocation, reinforcement learning, edge computing, Markov decision process

## Abstract

The rapid development of mobile device applications put tremendous pressure on edge nodes with limited computing capabilities, which may cause poor user experience. To solve this problem, collaborative cloud-edge computing is proposed. In the cloud-edge computing, an edge node with limited local resources can rent more resources from a cloud node. According to the nature of cloud service, cloud service can be divided into private cloud and public cloud. In a private cloud environment, the edge node must allocate resources between the cloud node and the edge node. In a public cloud environment, since public cloud service providers offer various pricing modes for users’ different computing demands, the edge node also must select the appropriate pricing mode of cloud service; which is a sequential decision problem. In this stydy, we model it as a Markov decision process and parameterized action Markov decision process, and we propose a resource allocation algorithm cost efficient resource allocation with private cloud (CERAI) and cost efficient resource allocation with public cloud (CERAU) in the collaborative cloud-edge environment based on the deep reinforcement learning algorithm deep deterministic policy gradient and P-DQN. Next, we evaluated CERAI and CERAU against three typical resource allocation algorithms based on synthetic and real data of Google datasets. The experimental results demonstrate that CERAI and CERAU can effectively reduce the long-term operating cost of collaborative cloud-side computing in various demanding settings. Our analysis can provide some useful insights for enterprises to design the resource allocation strategy in the collaborative cloud-side computing system.

## 1 Introduction

In recent years, the number of mobile devices, such as mobile phones, wearable devices, and sensors increased rapidly. Because of the limitations of computing power, memory, and battery capacity of mobile devices, it is usually unable to meet the requirements of the complex computing demands. To provide low latency and real-time services to mobile users, edge computing is proposed. Edge computing provides a virtual pool of configurable computing resources, and such resource instances are often referred to as virtual machines (VMs). Edge computing can provide users with low latency, location awareness, and high-quality service Mao et al. (2017); Weisong et al. (2019); Shi et al. (2016). However, edge nodes usually do not exhibit enough storage and computing resources when processing massive mobile device data. Therefore, collaborative cloud-edge computing has been proposed. To provide users with computing services, cloud and edge nodes cooperate with each other Chang et al. (2014).

In the collaborative cloud-edge computing, the edge node with limited local resources can rent more resources from the cloud node and pay corresponding costs to meet users’ demands. Because the computing cost of edge nodes changes dynamically according to their workload, if no strategic resource allocation exists when collaborative cloud-edge computing provides service, the computing resources in edge nodes with a relatively low computing cost cannot be used reasonably, and its computing cost will increase. Therefore, reducing the long-term operation cost on the premise of meeting the dynamic computing demands of users is a key problem in the collaborative cloud-edge computing.

In the real environment, cloud service can be divided into public cloud and private cloud according to their characteristics. In the private cloud, when the users’ computing demands randomly reach the edge node, the edge node needs to decide how to reasonably allocate resources between the cloud and edge nodes to satisfy users’ demands. In the public cloud, cloud service providers offer various pricing modes for cloud service, so the edge node also needs to select appropriate pricing mode of cloud service for collaborative computing according to users’ demands. In this paper, we will analyze how to allocate resources efficiently to reduce the long-term operation cost in the cloud-edge computing system to satisfy the dynamic demands of users under different cloud services. Since it is a sequential decision-making problem, we propose two resource allocation algorithm Cost Efficient Resource Allocation with private cloud (CERAI) and Cost Efficient Resource Allocation with public cloud (CERAU) based on deep reinforcement learning algorithms, deep deterministic policy gradient (DDPG) and P-DQN. Furthermore, we ran experiments to evaluate our algorithm against three typical resource allocation algorithms based on synthetic data and real data of Google dataset. The experimental results show that the algorithm proposed in this paper can achieve the lowest operation cost under different strength of demanding amount and computing time duration.

The structure of this paper is as follows. In Section 2, we introduce the related work. In Section 3 , we describe the basic settings. In Section 4, we model the problem as a parameterized action Markov decision process and introduce the resource allocation algorithm CERACE based on P-DQN. Next, we run experiments to evaluate the proposed algorithm in Section 5 and conclude the paper in Section 6.

## 2 Related Work

A lot of works about resource allocation in the cloud computing exists, such as Mao and Humphrey (2011); Abrishami et al. (2013); Malawski et al. (2015); Rodriguez and Buyya (2014). In Zhan et al. (2015), Zhan et al. did a deep survey about resource allocation in the cloud computing. Furthermore, some works about resource allocation in the edge computing also exist. Zhang et al. Zhang et al. (2019) used a Stackelberg game based approach to solve the multi-user offloading problem when the edge computing resource is limited in order to reduce the energy consumption. Guo et al. Guo et al. (2016) proposed an optimal policy based on a Markov decision process for scenarios with high mobile device density. Zhao et al. Zhao et al. (2015) proposed an offloading strategy that maximizes the number of tasks served while satisfying the users’ latency requirements. Gross et al. Gross et al. (2020) proposed a dynamic cost model to minimize the total time consumed by IoT devices in the mobile edge computing environment.

Because of the development of collaborative cloud-edge system, works about resource allocation in the cloud-edge system also exist. Wang et al. Wang and Wang (2021) proposed an optimization strategy for computing resource allocation of massive IoHT devices in cloud-edge computing environment. Yuan et al. Yuan and Zhou (2020) designed a collaborative computation offloading and resource allocation algorithm to maximize the profit of systems and meet the response time constraint. Next, Lin et al. Lin and Shen (2016) proposed a lightweight system called CloudFog to improve the quality of service for the corresponding delay problem in the cloud-based entertainment game. Then, Shen et al. Shen et al. (2020) proposed a dynamic task unloading method DOM with minority game in cloud-edge computing to address the vehicle computing resource shortage in the Internet of vehicles. Zhao et al. Zhao et al. (2019) proposed a collaborative approach in the cloud-edge computing to offload tasks to automobiles in vehicular networks. Wang et al. Wang et al. (2017) proposed an online algorithm to dynamically allocate resources in the collaborative cloud-edge system. Jiao et al. Jiao et al. (2017) designed an online algorithm which decouples the original off-line problem by constructing a series of regularized subproblems to reduce the cost. Dinh et al. Dinh et al. (2020) considered a hybrid cloud-edge computing system where edge devices with limited local resources can rent more resources from cloud nodes. Wang et al. Wang and Li (2021) proposed a dynamic multi-winner incomplete information game to offload tasks and allocate resource for multiple end users.

To the best of our knowledge, existing works about resource allocation in the collaborative cloud-edge system usually did not consider the cost of cloud-side resource, and they only consider a simple pricing mode. Furthermore, users’ demands are usually dynamically arriving in the real world, and existing works usually consider how to allocate resources when users’ demands are known. In this paper, we analyze the resource allocation problem in the collaborative cloud-edge computing given users’ dynamic demands and various pricing modes of cloud service.

## 3 Basic Settings

In this section, first, we introduce how the collaborative cloud-edge system works, and then, we introduce the basic settings. In conclusion, we describe our problems. We list the key symbols used in this paper in Table 1.

**TABLE 1:** Key symbols.

Notation	Description
*T*	Total time slots
*D*_*t*_	Demand information submitted by the user in time slot *t*
*d*_*t*_	Number of VMs requested of *D*_*t*_
*l*_*t*_	Computing time duration of *D*_*t*_
*E*	The total number of VMs of the edge node
*e*_*t*_	The number of remaining VMs of edge node in time slot *t*
$d_{t}^{e}$	Number of VMs provided by edge node
$d_{t}^{c}$	Number of VMs provided by cloud node
*h*_*t*_	Resource allocation record for *D*_*t*_
*η*_*t*_	Number of VMs released by edge nodes in time slot *t*
*p*_*e*_	Standby cost of one VM in the edge node
*p*_*f*_	Computing cost of one VM in the edge node
*p*_*c*_	Unit cost of VMs in private cloud
*p*_*upfront*_	Customization price of reserved instance in public cloud
*p*_*od*_	Unit cost of on-demand instance in public cloud
*p*_*re*_	Unit cost of reserved instance in public cloud
*p*_*t*_	Unit cost of spot instance in public cloud in time slot *t*

### 3.1 The Collaborative Cloud-Edge Environment

**FIGURE 1:**
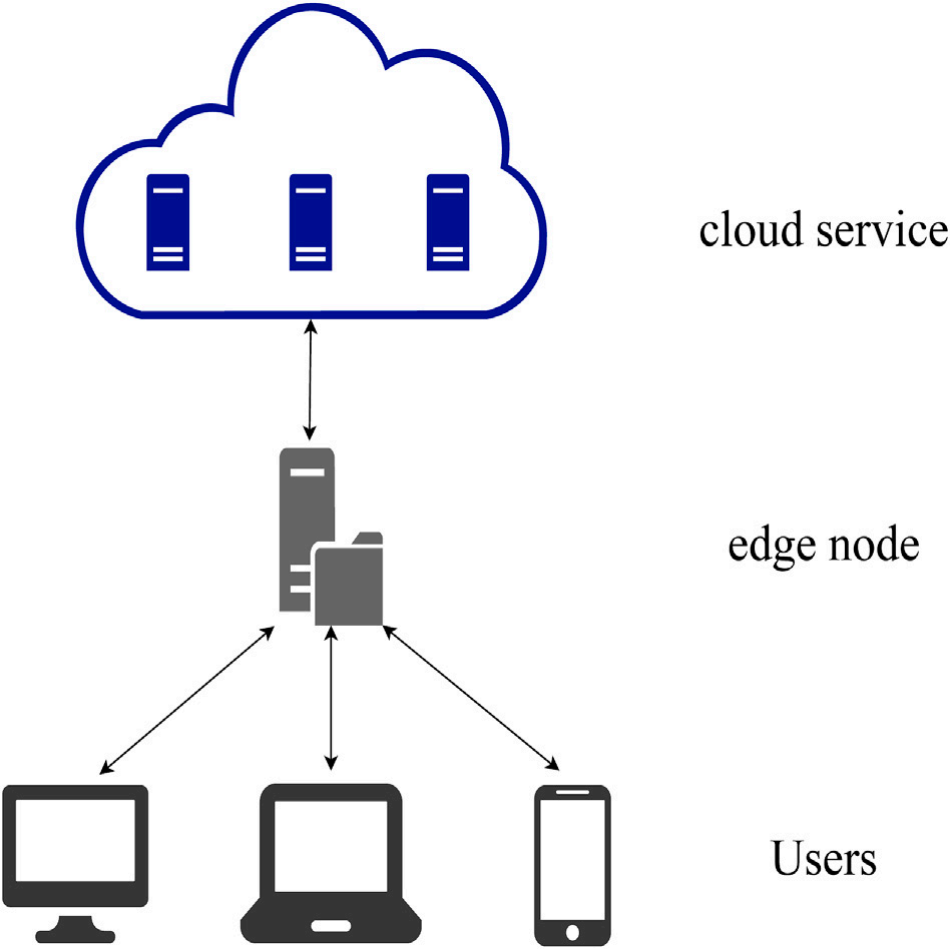
Collaborative cloud-edge computing system.

We consider the resource allocation problem in the multi-level collaborative computing environment Ren et al. (2019); Dinh et al. (2020) of “user-edge-cloud,” as shown in Figure 1. Our research focuses on the resource allocation strategy under collaborative cloud-edge. Therefore, in order to simplify the problem model, our model includes a single cloud node and an edge node, which can also be extended the setting with multiple edge nodes. According to the character of a cloud service, the cloud service can be divided into private cloud and public cloud.

In a private cloud environment, the edge node exhibits its own VMs to process users’ demands. However, because the number of VMs requested by the user may exceed the edge node’s capacity, the edge node needs to rents VMs from the cloud node to scale up its capacity. However, the cost of private cloud changes dynamically according to its physical computing cost, so the edge node needs to dynamically allocate resources at each time slot according to its resource allocation policy. After the edge node allocates resources through its policy, the computing cost of the edge node and private cloud in this time slot can be calculated and then transfer to the next time slot to receive new computing tasks.

In a public cloud environment, different from the private cloud, the public cloud provides a variety of cloud service pricing modes according to the demand characteristics of different demands. When using the cloud services of the public cloud, you need to pay according to the pricing mode. Cloud service providers such as Amazon, Microsoft, and Alicloud provided three different pricing modes^1^, each of which exhibits a different cost structure. Edge node needs to select appropriate pricing mode of cloud service and allocates users’ demands to either rented VMs or its own VMs.

### 3.2 User Setting

As described in Section 3.1, the time is discretized into *T* time slots. We assume that in each time slot *t*, the demand submitted by the user can be defined as the following:

$$D_{t}=\left(d_{t},l_{t}\right)$$ (1)

*D*_*t*_ is a pair of *d*_*t*_ and *l*_*t*_, where *d*_*t*_ is the number of VMs requested of *D*_*t*_, and *l*_*t*_ is the computing time duration of *D*_*t*_.

### 3.3 Computing Resources and Cost of Edge Nodes

The total computing resources owned by the edge node are represented by *E*. As the resource is allocated to users, we use *e*_*t*_ to represent the number of remaining VMs of edge node in time slot *t*. The number of VMs provided by the edge node is expressed as $d_{t}^{e}$ . The number of VMs provided by the cloud node is expressed as $d_{t}^{c}$ . It should be noted that if the edge node exhibits no available resources, it will hand over all the arriving computing tasks to the cloud service for processing. Now, we demonstrate the following:

$$d_{t}^{e}=\left\{\begin{array}{c} d_{t}-d_{t}^{c},e_{t}\geq 0\\ 0,e_{t}=0 \end{array}\right.$$ (2)

When the resource allocation can be successfully performed on the edge node, each demand processed by the edge node will generate an allocation record:

$$h_{t}=\left(d_{t}^{e},l_{t}\right)$$ (3)

which consists of two parts, where $d_{t}^{e}$ is the number of VMs provided by the edge node in this allocation, and *l*_*t*_ is the remaining computing time of this demand. When a new demand arrives and resource allocation is completed, an allocation record will be generated and added to an allocation record list:

$$H=<h_{1},h_{2},\ldots ,h_{m}>$$ (4)

At the end of each time slot, the edge node traverses these *m* records in the allocation record list *H* and then subtract one from the *l*_*i*_ in its record *h*_*i*_. If the remaining computing time of an allocation record is 0, it means that the demand has been completed. The edge node needs to release the corresponding VMs according to its record and delete the allocation record from the list. The number of VMs that completed the computing task and are waiting to be released at the end of time slot *t* is defined as *η*_*t*_. Then, we demonstrate the following:

$$\begin{array}{c} \eta_{t}=\overset{m}{\underset{i=1}{\sum }}d_{i}^{e}\\ s.t.l_{i}=0,h_{i}\in H \end{array}$$ (5)

Furthermore, the number of remaining VMs of the edge node at the next time slot *t* + 1 is the number of remaining VMs at the beginning of the time slot *t* minus the quantity allocated in the end of time slot *t* plus the quantity released because of the completion of the computing task in the time slot *t*. Then, the number of remaining VMs of the edge node at the time slot *t* + 1 is the following:

$$e_{t+1}=e_{t}-d_{t}^{e}+\eta_{t}$$ (6)

Note that in order to quickly respond to users’ computing demands, even if no computing demand is found, the machine still exhibits standby cost. Therefore, we consider that the cost of edge nodes consists of standby cost and computing cost. The standby cost of one VM in the edge node is *p*_*e*_. The computing cost of one VM in the edge node is *p*_*f*_. Now, the cost of the edge node in the time slot *t* is the following:

$$C_{t}^{e}=e_{t}p_{e}+\left(E-e_{t}\right)p_{f}$$ (7)

*e*_*t*_ *p*_*e*_ is the standby cost of the edge node in the time slot *t*, and (*E* − *e*_*t*_)*p*_*f*_ is the computing cost of the edge node.

### 3.4 Cost of Collaborative Cloud-Side Computing in Private Cloud

In time slot *t*, the cost of collaborative cloud-edge in private cloud environment is the following:

$$C_{t}^{pri}=d_{t}^{c}p_{c}+C_{t}^{e}$$ (8)

$d_{t}^{c}$ is the number of VMs provided by cloud node, *p*_*c*_ is the unit cost of VMs in private cloud, and $C_{t}^{e}$ is the cost of the edge node.

### 3.5 Cost of Collaborative Cloud-Side Computing in Public Cloud

In time slot *t*, the cost of collaborative cloud-edge in public cloud environment includes the computing cost of cloud nodes and the cost of edge node, which is the following:

$$\begin{array}{c} C_{t}^{pub}=X_{1}p_{od}d_{t}^{c}+X_{2}p_{\mathrm{upfront}}+X_{3}p_{re}d_{t}^{c}+X_{4}p_{t}d_{t}^{c}+C_{t}^{e}\\ X_{i}=\left\{\begin{array}{c} 1,The\;service\;is\;used\;\\ 0,The\;service\;is\;not\;used\; \end{array}\right. \end{array}$$ (9)

$X_{1}p_{od}d_{t}^{c}$ is the cost of on-demand instance, and *p*_*od*_ is the unit cost of on-demand instance. $X_{2}p_{\mathrm{upfront}}+X_{3}p_{re}d_{t}^{c}$ is the cost of reserved instance, where *p*_*upfront*_ is the customization price of reserved instance, and *p*_*re*_ is the unit cost of reserved instance. $X_{4}p_{t}d_{t}^{c}$ is the cost of spot instance, where *p*_*t*_ is the unit cost of spot instance, which is dynamically set by the cloud service provider. $C_{t}^{e}$ is the cost of the edge node.

### 3.6 Problem Formulation

We divide the whole time into *T* time slots. At the beginning of each time slot *t*, the user submits its demand to the edge node. Once receiving it, the edge node allocates demands to either cloud VMs or its own VMs according to its resource allocation strategy. In a public cloud environment, the edge node additionally determines the type of cloud service to be used. According to the allocation and the price of the corresponding cloud service set by the cloud service provider, the cost *C*_*t*_ of the current time slot *t* can be calculated, and then, the system enters the next time slot. Since the system will run for multiple time slots, we intend to minimize the long-term cost of the system $\sum_{t=1}^{T}C_{t}$ .

## 4 Resource Allocation Algorithm

In this section, we first describe the Markov Decision Process and the parameterized action Markov decision process, and then, we introduce two resource allocation strategy in collaborative cloud-edge environment based on DDPG and P-DQN.

### 4.1 Markov Decision Process

Referring to the work in Chen et al. (2021b), first, we need to model the collaborative cloud-side computing scenario. Given users’ dynamical demands over the time, the resource allocation problem is a sequential decision-making problem, which can be modeled as a Markov decision process. Markov decision process is a tuple (*S*, *A*, *P*, *r*, *γ*), where *S* is the finite set of states, *A* the finite set of actions, *P* is the probability of state transition, *r* and *γ* are the immediate reward and discount factor, respectively. Now, we introduce them in the following details.

• *s*_*t*_ = (*e*_*t*_, *η*_*t*−1_, *D*_*t*_, *p*_*c*_) ∈ *S* is used to describe the state of the edge node at the beginning of each time slot, where *e*_*t*_ is the number of remaining VMs of the edge node in *t*, *η*_*t*−1_ is the number of VMs returned in the previous time slot, *D*_*t*_ is the user’s demand information in *t*, and *p*_*t*_ is the unit cost of VMs in private cloud in *t*.
• *a*_*t*_ = (*x*_*e*_, *x*_*k*_) ∈ *A*, where *x*_*e*_ is the ratio of the number of VMs provided by the edge node to the total number of VMs. Also, *x*_*k*_ is the ratio of the number of VMs provided by the cloud node to the total number of VMs.
• $r_{t}=-C_{t}^{pri}$ is the reward in each time slot. Note that we want to reduce the long-term operation cost $R=\sum_{i=1}^{T}r\left(s_{i},a_{i}\right)$ ; therefore, the reward function is set as a negative value of the cost.

### 4.2 Parameterized Action Markov Decision Process

In the public cloud environment, first, the edge node needs to select the pricing mode of cloud service to be used and then determine the resource segmentation between the edge node and the cloud node in each time slot *t*. The resource allocation action can be described by parametric action. In order to describe this parameterized action sequential decision, parameterized action Markov decision process (PAMDP) Masson et al. (2016) is used.

Similar to Markov decision process, PAMDP is a tuple (*S*, *A*, *P*, *r*, *γ*). The difference with the Markov decision process is that *A* is the finite set of parameterized actions. The specific modeling is as follows.

• *s*_*t*_ = (*e*_*t*_, *η*_*t*−1_, *D*_*t*_, *p*_*t*_, *ξ*_*t*_) ∈ *S,* where *p*_*t*_ is the unit cost of spot instance in *t*, and *ξ*_*t*_ is the remaining usage time of reserved instance. When the edge node does not use this type of cloud service or it expires, this value is 0.
• *a*_*t*_ = (*x*_*e*_, (*k*, *x*_*k*_)) ∈ *A*, where *K* = {*k*_1_, *k*_2_, *k*_3_} is the set of all discrete actions, *k*_1_ is the on-demand instance, *k*_2_ is the reserved instance, and *k*_3_ is the spot instance.
• $r_{t}=-C_{t}^{\mathrm{pub}}$ is the reward in each time slot.

### 4.3 Resource Allocation Based on Deep Deterministic Policy Gradient

Reinforcement learning Kaelbling et al. (1996) has been widely used to solve the sequential decision-making problems. When faced with large or continuous state space, conventional reinforcement learning suffers from “curse of dimensionality.” In view of the widespread use of deep learning Huang et al. (2021); Jiang et al. (2021a,b); Huang et al. (2022), DeepMind combined deep learning with reinforcement learning and proposed deep reinforcement learning (DRL). In the collaborative cloud-side environment under the private cloud, the state space and the action space is a continuous space. Therefore, we use DDPG Lillicrap et al. (2015) to solve the resource allocation.

The DDPG algorithm is the classical algorithm of the Actor-Critic algorithm, where the Actor generates actions based on policies and interacts with the environment, while Critic evaluates Actor’s performance through a value function that guides Actor’s next action, thus improving its convergence and performance.

DDPG introduces the idea of DQN and contains four networks, where the main Actor network selects the appropriate action *a,* according to the current state, *s* and interacts with the environment:

$$a=\pi_{\theta }\left(s\right)+N$$ (10)

where N is the added noise. For the Critic master network, the loss function is the following:

$$\nabla J\left(w\right)=\frac{1}{m}\overset{m}{\underset{j=1}{\sum }}{\left(y_{j}-Q\left(\phi \left(s_{j}\right),a_{j},\omega \right)\right)}^{2}$$ (11)

where *y*_*j*_ is the target Q value and is calculated as the following:

$$y_{j}=r_{j}+\gamma Q^{\prime }\left(\phi \left(s_{j}^{\prime }\right),\pi_{\theta }\left(\phi \left(s_{j}^{\prime }\right)\right),\omega^{\prime }\right)$$ (12)

For the Actor master network, the loss function is the following:

$$\nabla J\left(\theta \right)=\frac{1}{m}\overset{m}{\underset{j=1}{\sum }}\left[{\left.{\left.\nabla_{a}Q\left(s_{i},a_{i},\omega \right)\right|}_{s=s_{i},a=\pi_{\theta }\left(s\right)}\nabla_{\theta }\pi_{\theta }\left(s\right)\right|}_{s=s_{i}}\right]$$ (13)

The parameters *ω* of the Actor target network and the parameters *θ* of the Critic target network are updated using a soft update:

$$\begin{array}{cc} \omega^{\prime } & \leftarrow \tau \omega +\left(1-\tau \right)\omega^{\prime }\\ \theta^{\prime } & \leftarrow \tau \theta +\left(1-\tau \right)\theta^{\prime } \end{array}$$ (14)

**FIGURE 2:**
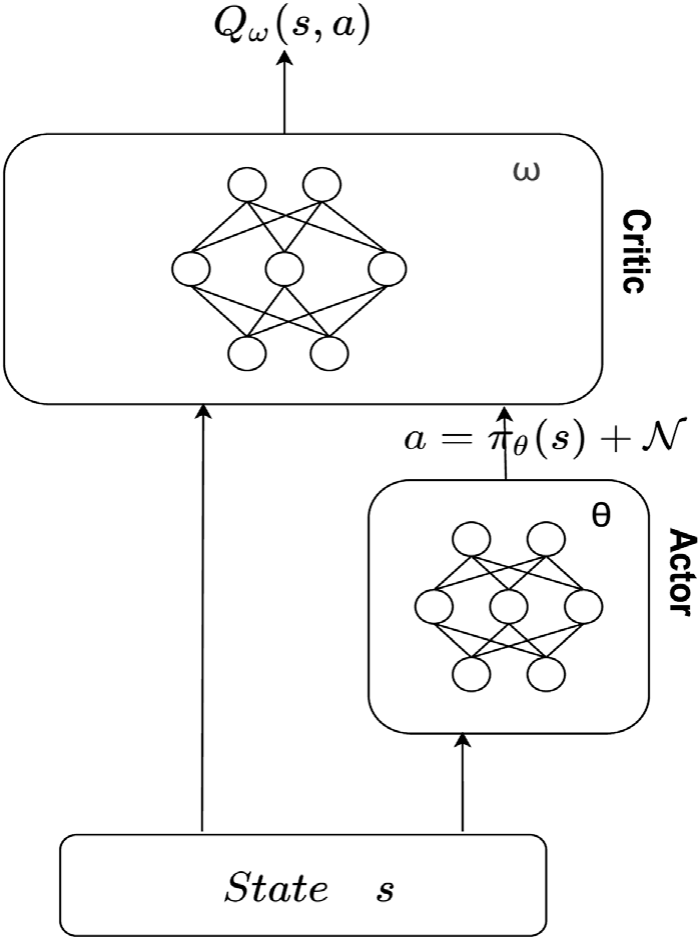
Structure of deep deterministic policy gradient (DDPG).

DDPG structure is shown in Figure 2 and the CERAI algorithm is shown in Algorithm 1. The input of the algorithm contains information about the user requests demands *D*_*t*_ and the unit cost of VMs in private cloud *p*_*c*_. At the beginning of each iteration of the algorithm, the edge node first needs to obtain the state *s*_*t*_ of the collaborative cloud-edge environment and then pass the state as the input of the neural network into the main Actor network to obtain the action *a*_*t*_. After the edge node gets the action, the number of demands to be processed by the edge node and the number of demands to be processed by the private cloud will be calculated by the action value, i.e., $d_{t}^{e}$ and $d_{t}^{c}$, respectively. Then, interaction with the environment based on $d_{t}^{e}$ and $d_{t}^{c}$, to get the next state, reward, and termination flag. Storing this round of experience to the experience replay pool, CERAI will sample from the experience replay pool and calculate the loss functions of Actor and Critic to update the parameters of the master and target networks. After one round of iterative, the training will be continued to the maximum number of training rounds set to ensure the convergence of the resource allocation policy.

Algorithm 1 Cost efficient resource allocation with private cloud (CERAI).

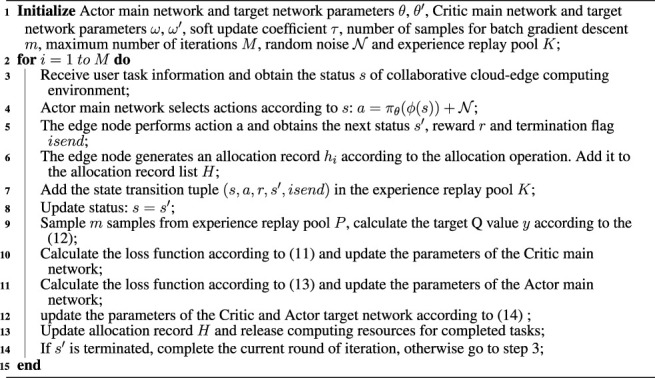

### 4.4 Resource Allocation Based on P-DQN

In the public cloud environment, the edge node needs to select the pricing mode of cloud service to be used and then determine the number of VMs to be rented from the cloud node. This is a mixture of discrete action space and continuous action space. Therefore, we use P-DQN Xiong et al. (2018) to solve the resource allocation.

The basic idea of P-DQN is as follows. For each action *a* ∈ *A* in the parametric action space, because of *x*_*e*_ + *x*_*k*_ = 1, we can only consider *k* and *x*_*k*_ in the action value function, that is *Q* (*s*, *a*) = *Q* (*s*, *k*, *x*_*k*_), where *s* ∈ *S*, *k* ∈ *K* is the discrete action selected in the time slot *t*, and *x*_*k*_ ∈ *X*_*k*_ is the parameter value corresponding to k. Similar to DQN, deep neural network *Q* (*s*, *k*, *x*_*k*_; *ω*) is used in P-DQN to estimate *Q* (*s*, *k*, *x*_*k*_), where *ω* is the neural network parameter. In addition, for *Q* (*s*, *k*, *x*_*k*_; *ω*), P-DQN uses the determined policy network $x_{k}\left(\cdot ;\theta \right):S\to X_{k}$ to estimate the parameter value $x_{k}^{Q}\left(s\right)$ , where *θ* is used to represent the policy network. That means the goal of P-DQN is to find the corresponding parameters *θ,* when *ω* is fixed. It can be written as the following:

$$Q\left(s,k,x_{k}\left(s;\theta \right);\omega \right)\approx Q\left(s,k,x_{k};\omega \right)$$ (15)

Similar to DQN, the value of *ω* can be obtained by minimizing the mean square error by gradient descent. In particular, step *t*, *ω*_*t*_ and *θ*_*t*_ are the parameters of value network and deterministic policy network, respectively. Then, *y*_*t*_ can be written as the following:

$$y=r+\underset{k\in \left[k\right]}{\max}Q\left(s^{\prime },k,x_{k}\left(s^{\prime },\theta_{t}\right);\omega_{t}\right)$$ (16)

where *s*′ is the next state after taking the mixed action *a* = (*k*, *x*_*k*_). The loss function of value network can be written as the following:

$$l^{Q}\left(\omega \right)=\frac{1}{2}{\left[Q\left(s,k,x_{k};\omega \right)-y\right]}^{2}$$ (17)

In a similar manner, the loss function of a policy network can be written as the following:

$$l^{\Theta }\left(\theta \right)=-\overset{K}{\underset{k=1}{\sum }}Q\left(s,k,x_{k}\left(s;\theta \right);\omega \right)$$ (18)

**FIGURE 3:**
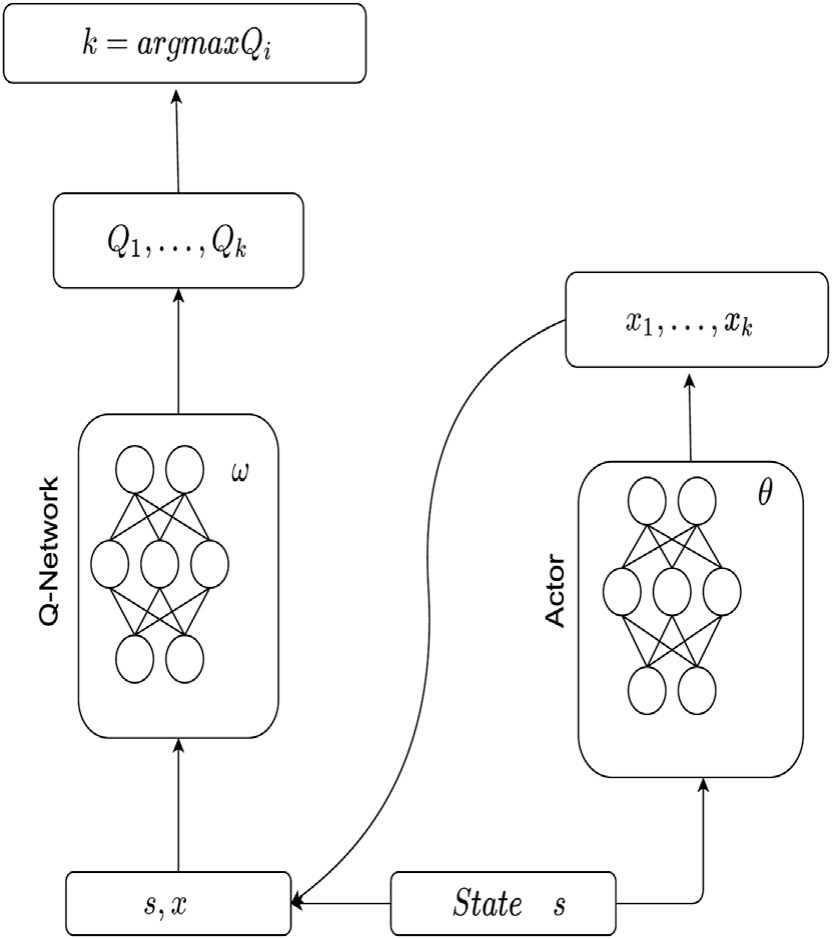
Structure of P-DQN.

P-DQN structure is shown in Figure 3. Now, we propose the resource allocation algorithm based on P-DQN, which is called Cost Efficient Resource Allocation with public cloud (CERAU), as shown in Algorithm 2. The input of the algorithm contains information about the user requests demands *D*_*t*_ and the unit cost of spot instance in public cloud in time slot *t p*_*t*_. At the beginning of each iteration of the algorithm, the edge node first needs to obtain the state *s*_*t*_ of the collaborative cloud-edge environment and then pass the state as the input of the neural network into the strategy network to obtain the parameter values of each discrete action. After the edge node gets the action, it will select the appropriate public cloud instance type based on the discrete values in the action and determine the number of public cloud instances to be used based on the parameter values. Then, interaction with the environment occurs, to get the next state, reward, and termination flag. Storing this round of experience to the experience replay pool, CERAU will sample from the experience replay pool and calculate the gradient of the value network and the policy network. Then, it will update the parameters of the corresponding networks. After one round of iterative, to ensure the convergence of the resource allocation policy, the training will be continued to the maximum number of training rounds set.

Algorithm 2 Cost Efficient Resource Allocation with public cloud (CERAU).

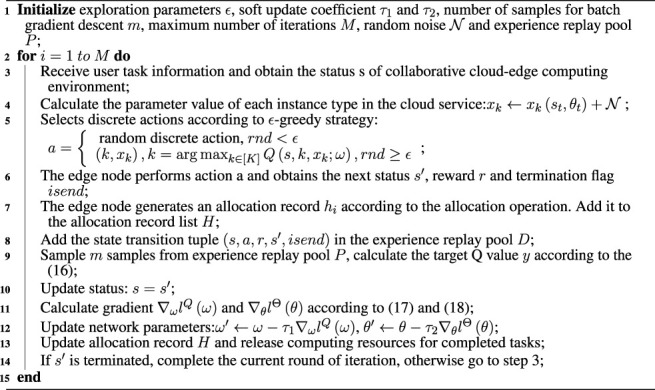

## 5 Experiment

### 5.1 Parameter Settings

The parameter settings used in this experiment are shown in Table 2. In this experiment, the initial number of VMs of edge nodes is set *E* = {60, 70, 80, 90, 100}. The service time of collaborative cloud-edge computing is 100 time slots, i.e., *T* = 100. The number of VMs and the computing time duration requested by users can be described by normal distribution Shao et al. (2006); Zhou and Chen (2008), i.e., $d_{t}∼N\left(\mu_{a},\sigma_{a}^{2}\right)$ , $l_{t}∼N\left(\mu_{l},\sigma_{l}^{2}\right)$ . The standby cost of one VM in the edge node is *p*_*e*_ = 0.03. The computing cost of one VM in the edge node is *p*_*f*_ = 0.2. The uniform distribution of the computing cost of private cloud is *p*_*c*_ ∼ *U* (1, 5). The unit cost of on-demand instance is *p*_*od*_ = 3.0. The customization price of reserved instance is *p*_*upfront*_ = 800. After the reserved instance is started, it can be used at a unit cost *p*_*re*_ = 1.5 within *T*_*r*_ = 20. The unit cost of spot instance is set by the cloud service provider. In this experiment, the assumption exists that its price fluctuation follows a normal distribution *p*_*t*_ ∼ *N* (1.5, 1). Because the spot instance is mainly aimed at the needs of small-scale and short-time computing tasks, we assume that only tasks with duration *T*_*m*_ = 6 or less can choose this type of instance.

**TABLE 2:** Parameter settings.

Notation	Description
*T* = 100	Total time slots
*E* = {60, 70, 80, 90, 100}	The total number of VMs of the edge node
$d_{t}∼N\left(\mu_{a},\sigma_{a}^{2}\right)$	The normal distribution of the number of VMs requested by the user
$l_{t}∼N\left(\mu_{l},\sigma_{l}^{2}\right)$	The normal distribution of the computing duration requested by the user
*p*_*e*_ = 0.03	Standby cost of one VM in the edge node
*p*_*f*_ = 0.2	Computing cost of one VM in the edge node
*p*_*c*_ ∼ *U* (1, 5)	The uniform distribution of the computing cost of private cloud
*p*_*od*_ = 3.0	Unit price of on-demand instance in public cloud
*p*_*upfront*_ = 800	Customization price of reserved instances in public cloud
*p*_*re*_ = 1.5	Unit price of reserved instance in public cloud
*T*_*r*_ = 20	Reserved duration of reserved instance in public cloud
*p*_*t*_ ∼ *N* (1.5, 1)	Normal distribution of unit price of spot instance
*T*_*m*_ = 6	Maximum service duration of spot instance
*γ* = 0.99	Discount factor
*τ* = 0.001	Soft update parameters of target network
*α*_1_ = 0.0001	Learning rate of Actor
*α*_2_ = 0.00001	Learning rate of Critic
*p* = 100,000	Size of experience pool
*m* = 128	Size of the batch sample in the experience pool

### 5.2 Experimental Dataset

In order to evaluate the performance of the algorithm under different initial states and different user demanding intensities, we run the experiments on the synthetic data and the realistic data on Google dataset, respectively.

First, we introduce the synthetic data. Similar to Dinh et al. (2020); Wang et al. (2014), we investigate the impact of different demanding intensities on the algorithm. The size of the demanding intensity is described by the mean and variance of the demanding instances in the normal distribution. The greater the mean and variance, the greater the demanding intensity. In particular, since the demand *D*_*t*_ consists of the number of VMs requested *d*_*t*_ and the computing time duration *l*_*t*_ (see Eq. 1), we consider the demanding intensity from the demanding amount and computing time duration, respectively. In more detail, in terms of the demanding amount *d*_*t*_, we consider three different groups of intensities:

• Group 1 is a low intensity group with normal distribution *d*_*t*_ ∼ *N* (5, 5^2^)
• Group 2 is a medium intensity group with normal distribution *d*_*t*_ ∼ *N* (10, 10^2^)
• Group 3 is a high intensity group with normal distribution *d*_*t*_ ∼ *N* (15, 15^2^)

where for each group, we assume that the computing time duration is at a medium level, *l*_*t*_ ∼ *N* (10, 10^2^). In terms of the computing time duration *l*_*t*_, we also consider three different groups of intensities:

• Group 1 is a low intensity group with normal distribution *l*_*t*_ ∼ *N* (5, 5^2^)
• Group 2 is a medium intensity group with normal distribution *l*_*t*_ ∼ *N* (10, 10^2^)
• Group 3 is a high intensity group with normal distribution *l*_*t*_ ∼ *N* (15, 15^2^)

where for each group, we assume that the demanding amount is at a medium level, *d*_*t*_ ∼ *N* (10, 10^2^).

**FIGURE 4:**
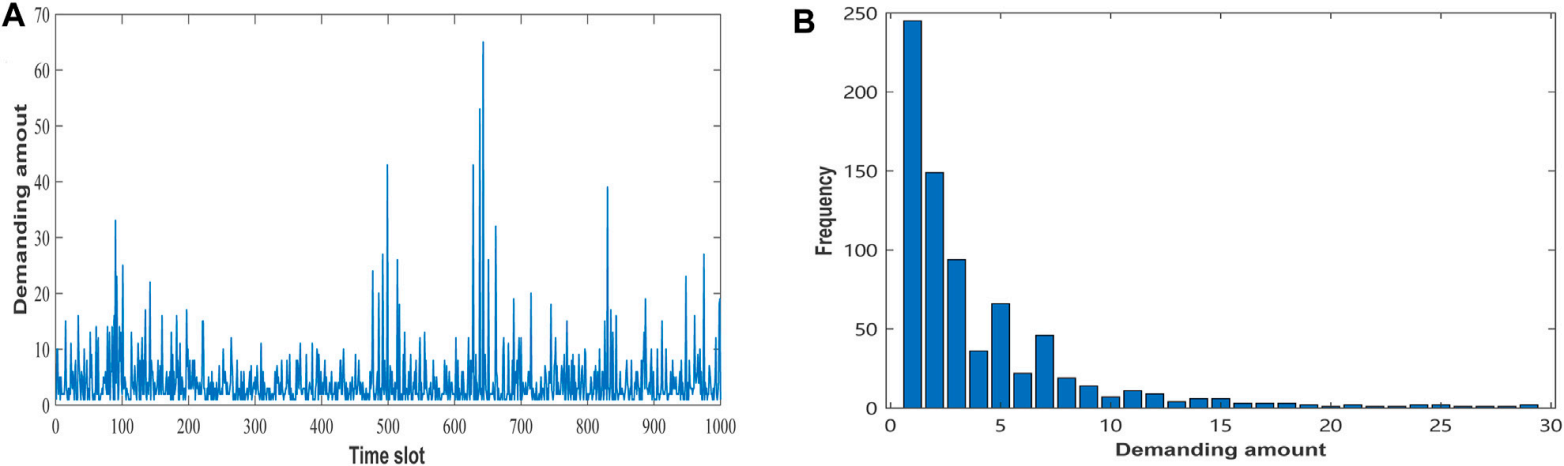
Demanding amount of Google dataset.

Also, we use Google cluster-usage traces data to further evaluate the proposed algorithm. Because no demanding information exists about computing time duration in this data, we assume that the computing time duration satisfies the normal distribution *l*_*t*_ ∼ *N* (10, 10^2^). Figure 4 show the fluctuation and frequency of user demands in Google dataset. As can be seen from Figure 4A, in the first 500 time slots, the demanding amount fluctuates less, while in the last 500 time slots, it fluctuates more. From Figure 4B, the demanding amount is mainly between 1 and 10, but some requests can reach 60 or more. According to this analysis of Google dataset, we set the initial capacity of the edge node in this experiment as *E* = {40, 50, 60, 70, 80}.

### 5.3 Benchmark Algorithms

First, we introduce the benchmark algorithms used in the private cloud environment. We evaluate our algorithm with three benchmark algorithms commonly used in existing collaborative cloud-side computing resource allocation works:

• EF (Edge First): this algorithm gives priority to the edge node to process user’s requests. Since the unit cost of edge nodes is lower than the unit cost of private clouds, this algorithm can be considered as a greedy algorithm.
• RANDOM: this algorithm randomly selects the edge node or the private cloud service. We add the random allocation algorithm to compare with the state-based resource allocation algorithm to show the strategy of the algorithm and its performance.
• Particle Swarm Optimization (PSO)Clerc (2010): PSO performs well on a wide range of optimization problems Liu et al. (2022). Unlike the above three resource allocation algorithms, PSO directly optimizes the resource allocation actions for each time slot to generate a resource allocation algorithm. That is to say, a sequential decision problem is transformed into a classical optimization problem.

Then, we introduce the benchmark algorithms used in the public cloud environment. Note that the existing similar works usually do not consider the various pricing modes of cloud service Wang et al. (2014); Champati and Liang (2015) in the public cloud. Therefore, we cannot evaluate our algorithm against these works. Instead, we evaluate our algorithm against the following three algorithms in terms of the long-term operation cost:

• E + O (Edge first + On demand): this algorithm gives priority to the edge node to process user’s requests. When the edge node demonstrates insufficient capacity to provide services, only the on-demand instance is used to process user’s requests.
• E + R (Edge + Random): this algorithm gives priority to the edge node to process user requests. When the capacity of the edge node is insufficient to provide computing services, the pricing mode of cloud service is randomly selected for collaborative computing.
• R + R (Random + Random): this algorithm randomly selects the pricing mode of cloud service and randomly determines the quantities for allocation.

### 5.4 Experimental Analysis for Private Cloud

#### 5.4.1 Impact of Demanding Amount on the Cost

**FIGURE 5:**
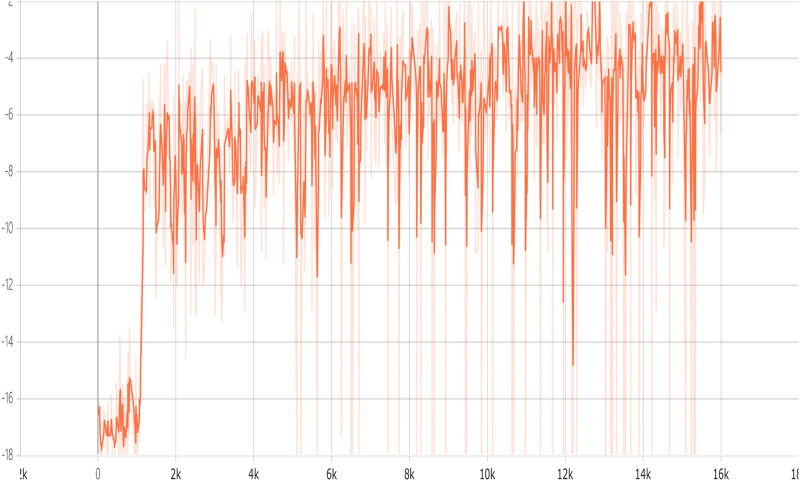
Accumulated reward versus training episodes of CERACI.

**FIGURE 6:**
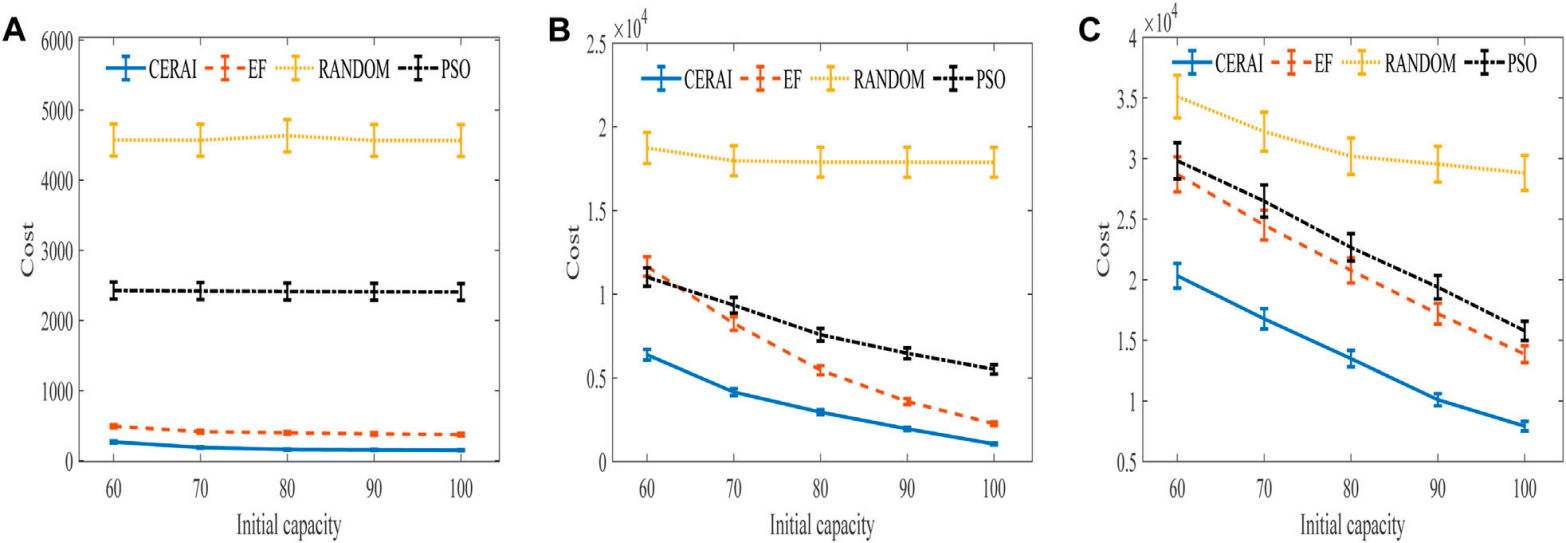
Experimental results of the impact of demanding amount on cost with private cloud. **(A)** Experimental results of low intensity group. **(B)** Experimental results of medium intensity group. **(C)** Experimental results of high intensity group.

We depict the training curve of CERAI in Figure 5, and the experimental results of the impact of demanding amount on the cost are shown in Figure 6. It can be seen that CERAI performs better than the other three algorithms under different request intensities.

In the low intensity group (Figure 6A), all algorithms vary little for different initial capacities of edge node, but the difference in cost required is large. The RANDOM algorithm demonstrates the highest cost, and the PSO demonstrates the second highest cost. This is because of the high dimensionality of the solution space of this problem and the lack of optimization capability when using PSO, which leads to its inability to search for the optimal solution. In contrast, the strategy-based algorithms EF and CERAI exhibit lower costs and show better performance in this group of experiments. CERAI updates its policy through continuous learning and iteration, which enables it to maintain a high performance, even when resources are abundant. CERAI reduces the cost by more than 45% compared to the suboptimal algorithm EF for different initial capacity of edge node.

In the medium intensity group (Figure 6B), it can be seen that RANDOM performance is still the worst, and it remains basically the same for different initial capacities of edge node. Next, the cost of EF, PSO, and CERAI decrease gradually as the initial capacity of the edge nodes rises. CERAI still shows the best performance in this group because CERAI does not use up all the resources of the edge node when user demand arrives, but it strategically reserves some of the resources for upcoming tasks. This results in cost savings by allowing the edge nodes to be used to handle the tasks when demanding amount is high.

In the high intensity group (Figure 6C), it can be seen that the cost of all algorithms decreases with the increase of the initial capacity of the edge node. The resources of the edge node are relatively scarce in this group of experiments, so private cloud nodes will be used more. Therefore, the gap between RANDOM and the other three algorithms is relatively reduced in this group compared to the other groups of experiments. The cost difference between the EF and the PSO is smaller because the EF strategy of prioritizing the use of edge nodes limits its performance in the presence of scarce resources of edge nodes. CERAI continues to show the best performance in this group of experiment.

#### 5.4.2 Impact of Computing Time Duration on the Cost

**FIGURE 7:**
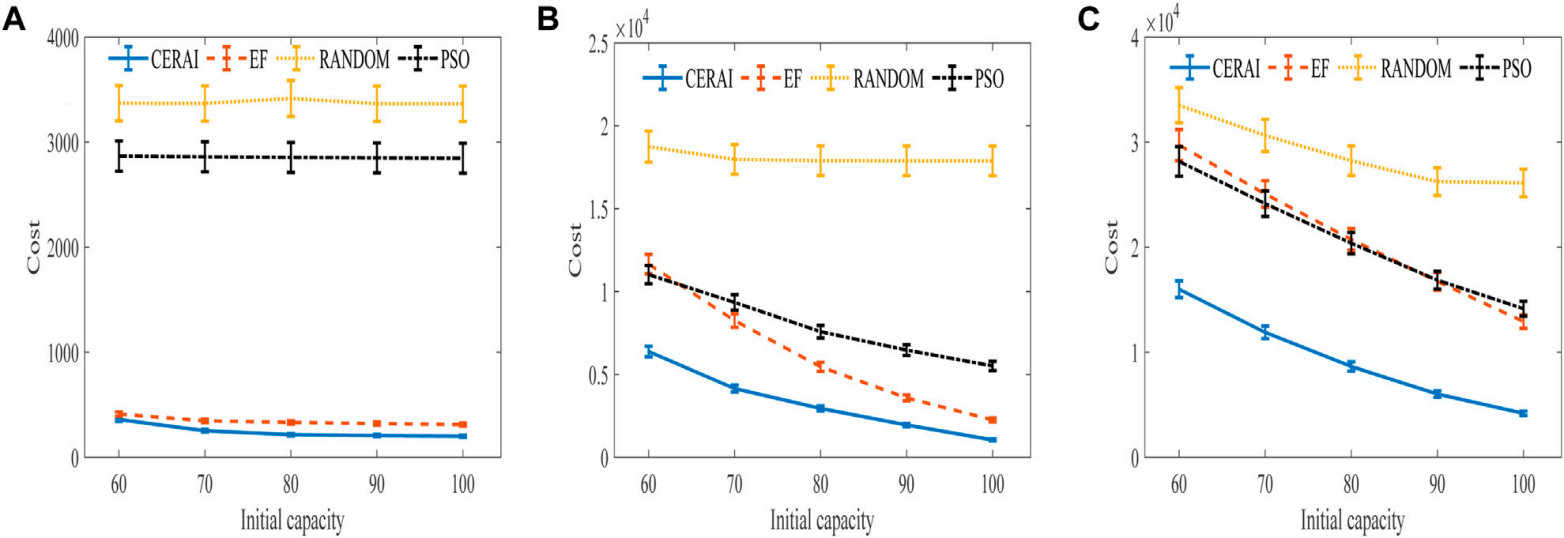
Experimental results of the impact of computing time duration on cost with private cloud. **(A)** Experimental results of low intensity group. **(B)** Experimental results of medium intensity group. **(C)** Experimental results of high intensity group.

The experimental results of the impact of computing time duration on the cost are shown in Figure 7. It can be seen that Figure 7A,B are almost the same as Figure 6A,B. The difference from the previous group is that in this group of high intensity experiments, PSO outperforms EF when the initial capacity of edge node is less. CERAI continues to show the best performance in this group of experiments. Also, it is worth noting that since the experimental parameters of the medium intensity group in both sets of experiments are identical, the experimental results are also identical.

#### 5.4.3 Experiment Based on Google Dataset

**FIGURE 8:**
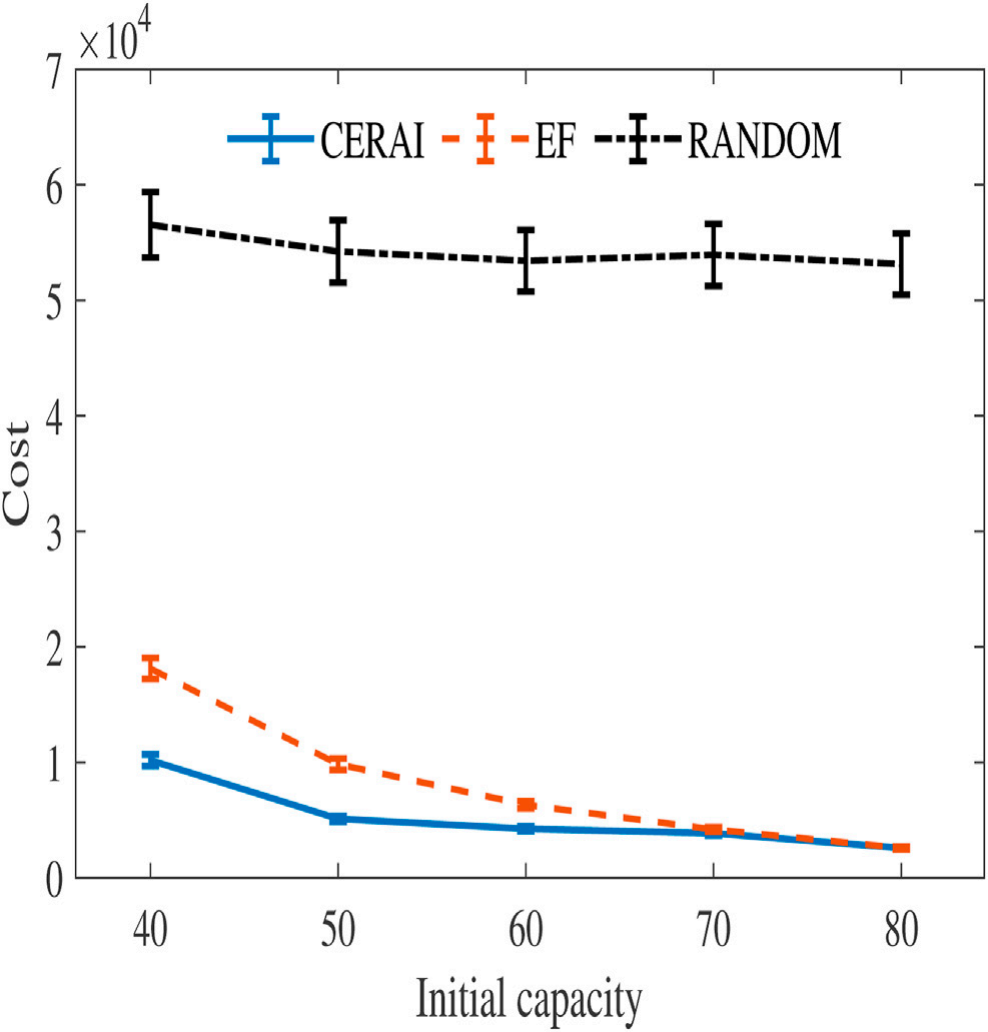
Experimental results based on Google dataset with private cloud.

In order to further evaluate the performance of the CERAI in the private cloud environment, we run the experiments on the Google dataset. The amount of data in the Google dataset experiment is large, and its solution space is too large for the PSO, so it cannot be solved using PSO. Therefore, the experiments in this group compare CERAI with EF and RANDOM. The experimental results are shown in Figure 8, and it can be seen that CERAI demonstrates a greater advantage over the EF when the initial resources of the edge node are small. The advantage of CERAI is weakened when the resources of edge node are relatively sufficient.

### 5.5 Experimental Analysis for Public Cloud

#### 5.5.1 Impact of Demanding Amount on the Cost

**FIGURE 9:**
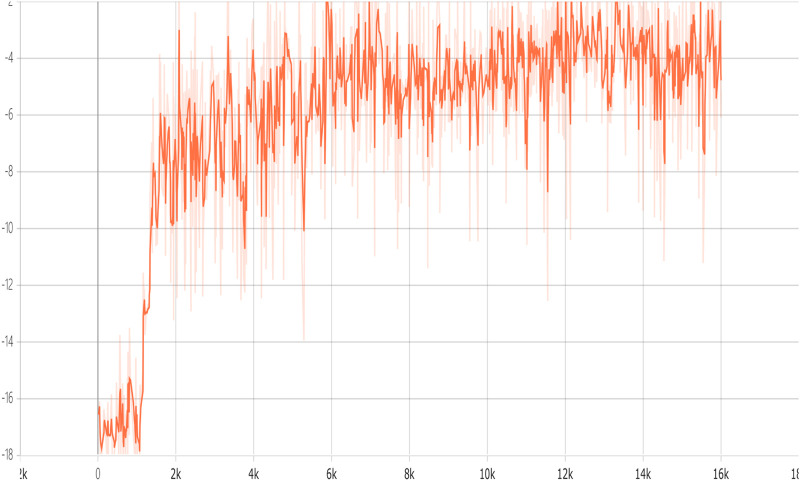
Accumulated reward versus training episodes of CERACU.

**FIGURE 10:**
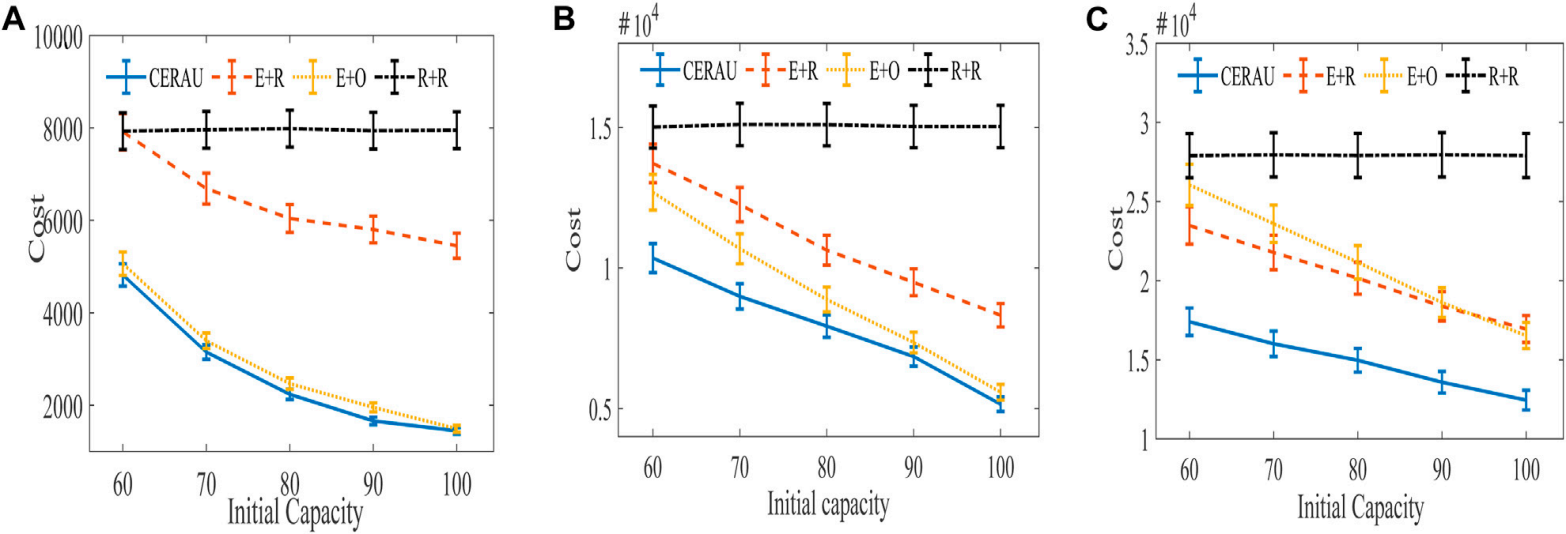
Experimental results of the impact of demanding amount on cost with public cloud. **(A)** Experimental results of low intensity group. **(B)** Experimental results of medium intensity group. **(C)** Experimental results of high intensity group.

The training curve of CERAU are shown in Figure 9, and the experimental results of the impact of demanding amount on the cost with public cloud are shown in Figure 10. It can be seen that CERAU performs better than the other three algorithms under different request intensities.

In the low intensity group (Figure 10A), R + R demonstrates little changes on the cost with respect to the different initial capacity of edge nodes. Its performance is the worst. The performance of E + R is the same as R + R when the initial capacity of edge nodes is 60. With the increase of the initial capacity of edge nodes, its cost decreases. CERAU and E + O demonstrate almost the same performance of the cost. This is because when users’ request intensity is low and the computing resources of edge nodes are relatively sufficient, E + O can also demonstrate a good performance on the cost. At this moment, no need to use reserved instances in cloud services is found. Since CERAU can use the spot instance with cheaper price than the on-demand instance, its performance is a little better than E + O.

In the medium intensity group (Figure 10B), our algorithm can still outperform other algorithms. We find that the cost of R + R under different initial capacity of edge nodes is still the highest. Next, the cost of the other three algorithms decreases gradually with the increase of the capacity of edge nodes. When the initial capacity is low, CERAU exhibits obvious advantages over the algorithm E+O.

In the high intensity group (Figure 10C), compared with other algorithms, the cost of CERAU can be reduced by more than 25% under different initial capacity of edge nodes. Compared to Figure 10B, we find that E + R perform a little better than E + O. This is because given high demand, the capacity of edge nodes is relatively scarce. E + O uses more on-demand instances; therefore, its cost is higher than E + R, which chooses instance type randomly.

#### 5.5.2 Impact of Computing Time Duration on the Cost

**FIGURE 11:**
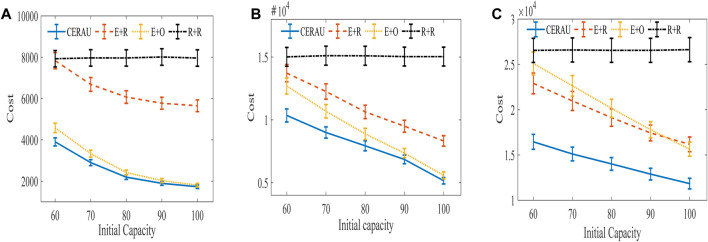
Experimental results of the impact of demanding amount on cost with public cloud. **(A)** Experimental results of low intensity group. **(B)** Experimental results of medium intensity group. **(C)** Experimental results of high intensity group.

The experimental results of the impact of computing time duration on the cost are shown in Figure 11. It can be seen that the results are similar to Figure 6. In more detail, Figure 11A,B are almost the same as Figure 10A,B. A slight difference is found between Figure 10(C) and Figure 11C. This shows that when the initial capacity of the edge node is insufficient and the user’s request intensity is low, CERAU is more sensitive to the change of the user’s demanding amount. At this time, CERAU can more effectively reduce the cost. When the user’s request intensity is high, CERAU demonstrates almost the same sensitivity to the user’s demanding amount and computing time duration, and we can effectively reduce the cost of the system. Similar to CERAI’s experiment, since the experimental parameters of the medium intensity group in both sets of experiments are identical, their experimental results are also identical.

#### 5.5.3 Experiment Based on Google Dataset

**FIGURE 12:**
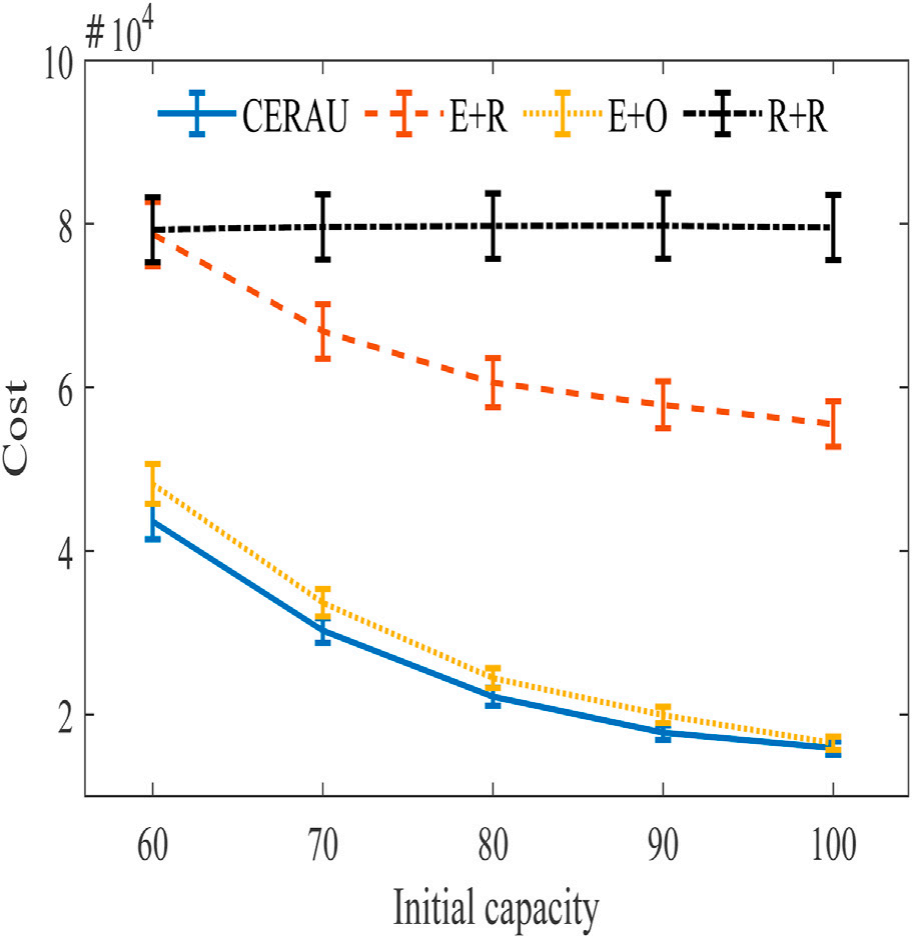
Experimental results based on Google dataset with public cloud.

The experimental results of the Google dataset are shown in Figure 12. The cost of the four algorithms under different initial capacity of edge nodes from high to low is R + R, E + R, E + O, and CERAU. The cost difference between E + O and CERAU is small. This is because the demanding amount in Google dataset is mainly between 1 and 10, which is similar to the experiment of low intensity group.

## 6 Conclusion

In this paper, we analyze resource allocation problem in the collaborative cloud-edge computing under private and public clouds, respectively. We model the problem as Markov decision process and PAMDP, and then, we propose the resource allocation algorithm CERAI and CERAU based on the DRL algorithm DDPG and P-DQN. In conclusion, we run experiments to evaluate our strategy against three typical algorithms on the synthetic data and the real data of Google dataset. The experimental results show that CERAI and CERAU can effectively reduce the long-term operation cost of collaborative cloud-side computing system in various settings. In this paper, we do not consider the cooperation of edge nodes. In the future, we want to extend the analysis to the case with more edge nodes, where edge nodes can cooperate with each other to accomplish the users’ tasks. This scenario can be solved by combining it with game theory by referring to the work in Chen et al. (2021a). For example, the edge node can use the idle resources of adjacent nodes for the collaborative computing.

## Data Availability

The original contributions presented in the study are included in the article/supplementary material, and further inquiries can be directed to the corresponding author.

## Footnotes

1 On-demand instance: This pricing mode allows users to pay with the fixed time granularity set by the platform, and the price is fixed in a long period of time. Reserved instance: this pricing mode requires the user to submit the reservation time in advance and pay the corresponding reservation fee to have the instance within the contract time. It is applicable to users with a large number of computing demands, and the unit price is usually approximately 50% lower than that of on-demand instances. Spot instance: the instance of this mode is obtained by bidding, and its price changes in different time slots, which is usually used for short-term computing demands.
